# 15‐Point versus pass/fail grading in orthodontic education: A randomized controlled trial

**DOI:** 10.1002/jdd.13815

**Published:** 2024-12-19

**Authors:** Marina Julia Bialas, Jonas Q. Schmid, Claudius Middelberg, Thomas Stamm, Moritz Blanck‐Lubarsch

**Affiliations:** ^1^ Department of Orthodontics University of Münster Münster Germany

**Keywords:** dental education, grading system, manual skills, orthodontic appliances, pass/fail

## Abstract

**Objectives:**

There is a lack of evidence on whether a grading system or a pass/fail system influences manual skills in dental education. This parallel‐group randomized controlled trial aimed to assess the influence of a 15‐point grading system compared with a pass/fail evaluation on the quality of orthodontic appliances in dental education.

**Methods:**

Predoctoral dental students of three orthodontic courses (*n* = 139) were randomly assigned to either the test group (15‐point grading system) or the control group (pass/fail) using sealed envelopes. In both groups, the fabricated orthodontic appliances were assessed by five calibrated dentists using standard criteria. The primary outcome was the quality of the orthodontic appliances using a 15‐point grading system. Group differences were evaluated with Mann‐Whitney *U* tests and Fisher ´s exact tests.

**Results:**

The quality of the orthodontic appliances was slightly higher in the test group (*n* = 68) compared with the control group (*n* = 70) in all three courses with mean grading values of 11.63 ± 0.75 versus 11.59 ± 0.99, 10.96 ± 0.83 versus 10.85 ± 0.82, and 10.93 ± 1.15 versus 10.14 ± 1.03. However, a statistically significant difference was found only in course 3 (*p* = 0.0222). Female participants performed better than males in all three courses (*p* = 0.0207).

**Conclusion:**

The implementation of a 15‐point grading system has a positive impact on the quality of appliances in orthodontic education and can be recommended. However, the differences were small and clinically meaningful in only one of the three courses evaluated.

## 1 INTRODUCTION

There are several ways to evaluate student performance in dental education. Whether to use a grading system or a pass/fail evaluation has been the subject of numerous studies.^1^, ^2^, ^3^, ^4^, ^5^, ^6^, ^7^, ^8^ To date, there is no scientific consensus on whether a grading or pass/fail system should be preferred.^1^

It was found that the use of a pass/fail system instead of a five‐interval grading system in the first 2 years of medical school did not result in a decline in the academic performance of students.^1^, ^9^ Several other studies support these findings.^3^, ^10^ In addition, a pass/fail system has been shown to significantly improve student satisfaction and well‐being.^1^, ^3^, ^8^, ^9^ However, pass/fail evaluation may not only increase well‐being but also facilitate intrinsic motivation and competency‐based education while reducing students’ stress and anxiety.^8^ Pass/fail systems have also been found to reduce competition and increase collaboration among students.^11^ While there seems to be a consensus that pass/fail systems increase students’ well‐being, the impact of pass/fail systems on students’ academic performance is less obvious.^5^

In contrast, grades seem to be useful in providing students with more detailed feedback on the quality of their performance and their strengths and weaknesses.^2^ A seven‐point grading system was found to be preferable to a pass/fail system, leading to higher students’ motivation and better performance than a pass/fail system.^6^ Some authors suggest a grading system because the “pass” category obscures the different levels of performance of individual students. When a grading system is used, it becomes easier to identify those students who have met the minimum standards for the task and may require additional support.^2^

There is a lack of evidence on whether a grading system or pass/fail system has an impact on the quality of manual skills in dental education as most studies have only examined the effect on theoretical knowledge. This study adds to the literature on this topic. Manual dexterity and technical skills are mandatory in dental education because dentistry is a technical discipline and good performance in dental education is more than just theoretical knowledge. The fabrication of orthodontic appliances seems to be well suited to practice a variety of basic technical skills and to develop competencies such as wire bending, acrylic processing, and polishing. These skills are not limited to orthodontics, which qualifies them to evaluate manual skills in dentistry. Therefore, the aim of this study was to assess the influence of a 15‐point grading system compared with a pass/fail evaluation on the quality of orthodontic appliances in dental education. The research hypothesis was that a 15‐point grading system affects the quality of the orthodontic appliances fabricated by dental students compared with a dichotomous pass/fail system.

## 2 MATERIALS AND METHODS

### 2.1 Trial design

This study was a single‐center two‐arm parallel group randomized controlled trial with a 1:1 allocation ratio. No changes were made to the trial after commencement.

### 2.2 Participants

Eligible participants were predoctoral dental students from three different orthodontic courses that are part of the clinical curriculum in the 6th, 7th, and 8th semesters. Inclusion criteria were (1) taking the course for the first time and (2) written informed consent to participate. Exclusion criteria were (1) repetition of a course, (2) violation of the course guidelines, (3) failure to complete the course, or (4) missing written informed consent to participate.

The study took place at the Department of Orthodontics, University of Münster, Germany, from April 2017 to July 2017. Permission to conduct this study was given by the Ethics Commission of the Medical Faculty of the University of Münster, Germany (2017‐216‐f‐S). Participation in the study was voluntary and all participants gave informed consent before participating.

### 2.3 Study protocol

Each student in both groups was provided with an iPad for the duration of the three orthodontic courses and was instructed to install the iTunes U e‐learning application (Apple Inc.). The control group received a conventional paper‐based dichotomous pass/fail evaluation sheet. This evaluation sheet had to be presented to the course supervisors (five dentists from the Department of Orthodontics) for each step of the orthodontic appliance fabrication (Table 1). If the result was sufficient, the participant received a signature on the evaluation sheet for passing as immediate feedback. Without the knowledge of the students, the results of the sufficient steps were also evaluated like those of the students in the test group using a 15‐point grading system and documented for later evaluation. In case the performance was not sufficient, the student had the chance to improve the steps to pass. A step was considered inadequate if the work was of insufficient quality or functionality and therefore did not receive even 4 out of 15 points, as 1 to 3 points were considered a failing performance.

**TABLE 1:** Intermediate steps in the manufacturing process that were evaluated by the supervisors for courses 1, 2, and 3.

Course	Appliance	Step 1	Step 2	Step 3	Step 4	Step 5	Step 6	Step 7	Step 8	Step 9	Step 10
**1**	Schwarz plate	Construction plan of elements	Adams clasps	Ball and Arrow clasps	Protrusion bow	Labial bow	Expansion screw	Model ready for resin	Appliance completed and polished		
**2**	Karwetzky's U‐bow activator type 1	Models inserted in fixator	Labial bow lower jaw	Labial bow upper jaw	Protrusion bow lower jaw	Protrusion bow upper jaw	Expansion screw	Models ready for resin	Appliance completely polished without U‐bow	U‐bow insertion	Appliance completed with U‐bow and polished
**3**	Fränkel's functional regulator type 3	Models inserted in fixator	Model reduction at upper jaw	Wax built up	Protrusion bow upper jaw	Palatal connector upper jaw	Connecting elements upper jaw	Labial bow lower jaw	Occlusal rests lower jaw	Appliance completed and polished	

For participants in the test group, a successful manufacturing step in the fabrication of the orthodontic appliance was confirmed by the course supervisors by entering a grade in iTunes U. A 15‐point grading system was used, with grades ranging from 1 to 15 points, with 15 points being the best score and 1 point being the worst score. As in the control group, they had the opportunity to improve their work if a partial step was insufficient. Students in the test group were able to check their individual grades in iTunes U immediately.

**FIGURE 1:**
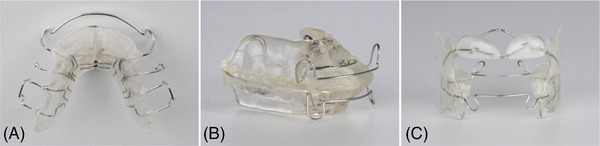
(A) Schwarz plate; (B) Karwetzky's U‐bow activator type 1; (C) Fränkel's functional regulator type 3 (FR‐3).

Three different orthodontic removable appliances were manufactured in the three courses (Figure 1). In course 1, a Schwarz Plate was manufactured for the upper jaw (Figure 1A). Karwetzky's U‐Bow Activator Type 1 was manufactured in course 2 (Figure 1B). In course 3, the Functional Regulator Type 3 according to Prof. Fränkel was manufactured (Figure 1C). For better comparability, all orthodontic appliances had to be manufactured in clear resin without color or glitter. For each appliance, intermediate steps in the manufacturing process were defined and evaluated by the supervisors (Table 1).

Five dentists (*f* = 2, *m* = 3) assessed the orthodontic appliances. Prior to the start of this study, these five course supervisors were calibrated to reduce the risk of bias and decided on binding criteria for the three orthodontic appliances to maximize interrater reliability. For this reason, videos of the ideal fabrication of these orthodontic appliances by a professional dental technician were viewed and a consensus was reached on the grading of the quality of the individual fabrication steps.

### 2.4 Outcomes

The primary outcome was the quality of the orthodontic appliances using a 15‐point grading system. A secondary outcome was the influence of gender on the quality of the appliances.

### 2.5 Sample size

A sample size calculation using G*Power 3.1 based on α = 0.05 and a power 1−β = 0.80 was performed. Assuming a clinically meaningful group difference in the quality of orthodontic appliances of 0.5 points (SD 1 point), an effect size (Cohen's *d*) of 0.5 was calculated, corresponding to a moderate effect. These values suggested that each group (15‐point grading system and pass/fail) required a minimum of 64 participants. Considering a potential dropout rate of 10%, the sample size was adjusted to approximately 70 participants per group.

### 2.6 Randomization sequence

Students were randomly assigned to the test and control groups. The randomization was carried out via the random allocation rule. For this purpose, all students drew a folded lot with a 0 or 1 from a ballot box without putting it back and were assigned to their respective groups.

### 2.7 Statistics

The evaluation of the collected data was carried out using the software IBM SPSS Statistics 26 (IBM Corp.). Interrater reliability was evaluated using intraclass correlation coefficients (ICC). For this purpose, all appliances in one randomly selected course were re‐evaluated by the calibrated investigators after at least 4 weeks. ICC estimates and their 95% confidence intervals were calculated based on an absolute‐agreement, two‐way mixed effects model. Interpretation of the correlation coefficients followed the cut‐off limits of Koo and Li, 2016.^12^ Inferential statistics are intended to be exploratory, not confirmatory, and should be interpreted accordingly. For the final evaluation of each course, the mean value was calculated for the corresponding participants over the grades of the 8–10 production steps. For the descriptive analysis, continuous variables using mean, standard deviation (SD), median, and range were reported. Box and whisker plots were used to visualize the results, and Mann‐Whitney *U* tests (or Kruskal‐Wallis tests) were used to compare the control and test groups. Categorical variables were described by absolute and relative frequencies and were compared between groups by Fisher´s exact test. *p* values less than 0.05 were considered to indicate a statistically significant difference. All reported *p*‐values were two‐sided.

## 3 RESULTS

**FIGURE 2:**
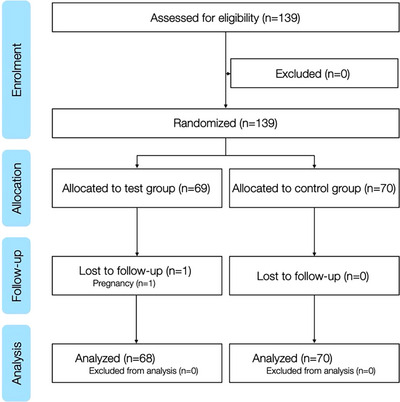
Flowchart of participants.

A flowchart of participants is shown in Figure 2. In total, 139 dental students were randomized in this study consisting of 90 female and 49 male students. The mean age of the students in all three courses was 24.2 (± 3.27) years. In course 1 the mean age was 23.7 (±4.03) years, in course 2 24.33 (±2.73) years, and in course 3 24.63 (± 2.44) years. There was a drop‐out of one female student at the beginning of course 2 due to pregnancy, reducing the total number of evaluable participants to 138. The baseline characteristics of the three groups are shown in Table 2. There was a significant (*p* = 0.0205) unequal distribution of female and male participants as shown in Table 3. Descriptive statistics for the quality of the orthodontic appliances using a 15‐point grading system are shown in Table 4.

**TABLE 2:** Distribution of participants in courses 1–3 by gender.

Course (appliance)	Participants total	Test group	Control group
1 (Schwarz plate)	*n* = 56 (39 female, 17 male)	*n* = 28 (15 female, 13 male)	*n* = 28 (24 female, 4 male)
2 (Karwetzky's U‐bow activator type 1)	*n* = 42 (23 female, 19 male)	*n* = 20 (9 female, 11 male)	*n* = 22 (14 female, 8 male)
3 (Fränkel's functional regulator type 3)	*n* = 40 (27 female, 13 male)	*n* = 20 (13 female, 7 male)	*n* = 20 (14 female, 6 male)
Total	*n* = 138 (89 female, 49 male)	*n* = 68 (37 female, 31 male)	*n* = 70 (52 female, 18 male)

**TABLE 3:** Distribution by gender and group.

	Gender		
	Male	Female	Total
Control group	18	52	70
Test group	31	37	68
Total	49	89	138

**TABLE 4:** Statistical comparison for the three courses and in total according to control group, test group, and overall.

	Total	Control group	Test group	*p*‐value
Course 1				
*n*	56	28	28	0.8761
Mean (SD)	11.61 (0.87)	11.59 (0.99)	11.63 (0.75)	
Median (Range)	11.69 (9.38–13.25)	11.69 (9.38–13.25)	11.63 (9.75–12.88)	
Course 2				
*n*	42	22	20	0.8203
Mean (SD)	10.90 (0.82)	10.85 (0.82)	10.96 (0.83)	
Median (Range)	11.0 (9.20–12.50)	11.00 (9.20–12.20)	11.00 (9.40–12.50)	
Course 3				
*n*	40	20	20	0.0222^*^
Mean (SD)	10.53 (1.15)	10.14 (1.03)	10.93 (1.15)	
Median (Range)	10.5 (8.44–13.44)	10.11 (8.63–12.44)	11.06 (8.44–13.44)	
In total				
*n*	138	70	68	0.1310
Mean (SD)	11.08 (1.04)	10.94 (1.11)	11.22 (0.96)	
Median (Range)	11.21 (8.44–13.44)	11.10 (8.63–13.25)	11.38 (8.44–13.44)	

^*^ *p* < 0.05.

Interrater reliability was good with an ICC of 0.826 (95% CI: 0.682–0.902). The quality of the orthodontic appliances was slightly higher in the test group compared with the control group in all three courses. Only in course 3, there was a statistically significant and clinically meaningful difference between the control and test groups (*p* = 0.0222) with a mean grading value of 10.14 ± 1.03 for the control group and a mean value of 10.93 ± 1.15 for the test group. There was no statistically significant difference between both groups in course 1 (*p* = 0.8761) with mean values of 11.59 ± 0.99 versus 11.63 ± 0.75 and in course 2 (*p* = 0.8203) with values of 10.85 ± 0.82 versus 10.96 ± 0.83.

**FIGURE 3:**
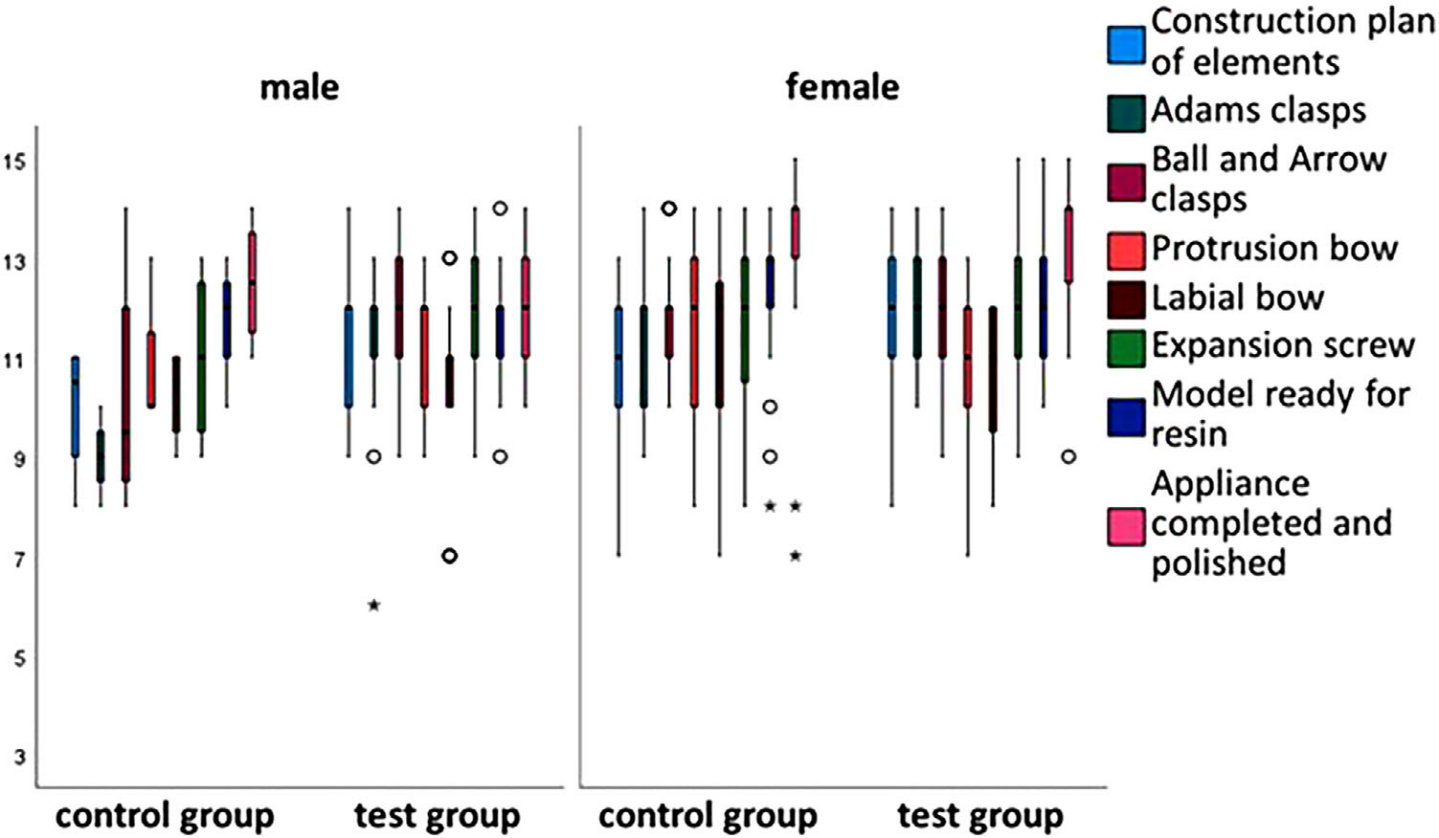
Grades of the Schwarz plate fabricated by the students in course 1.

**FIGURE 4:**
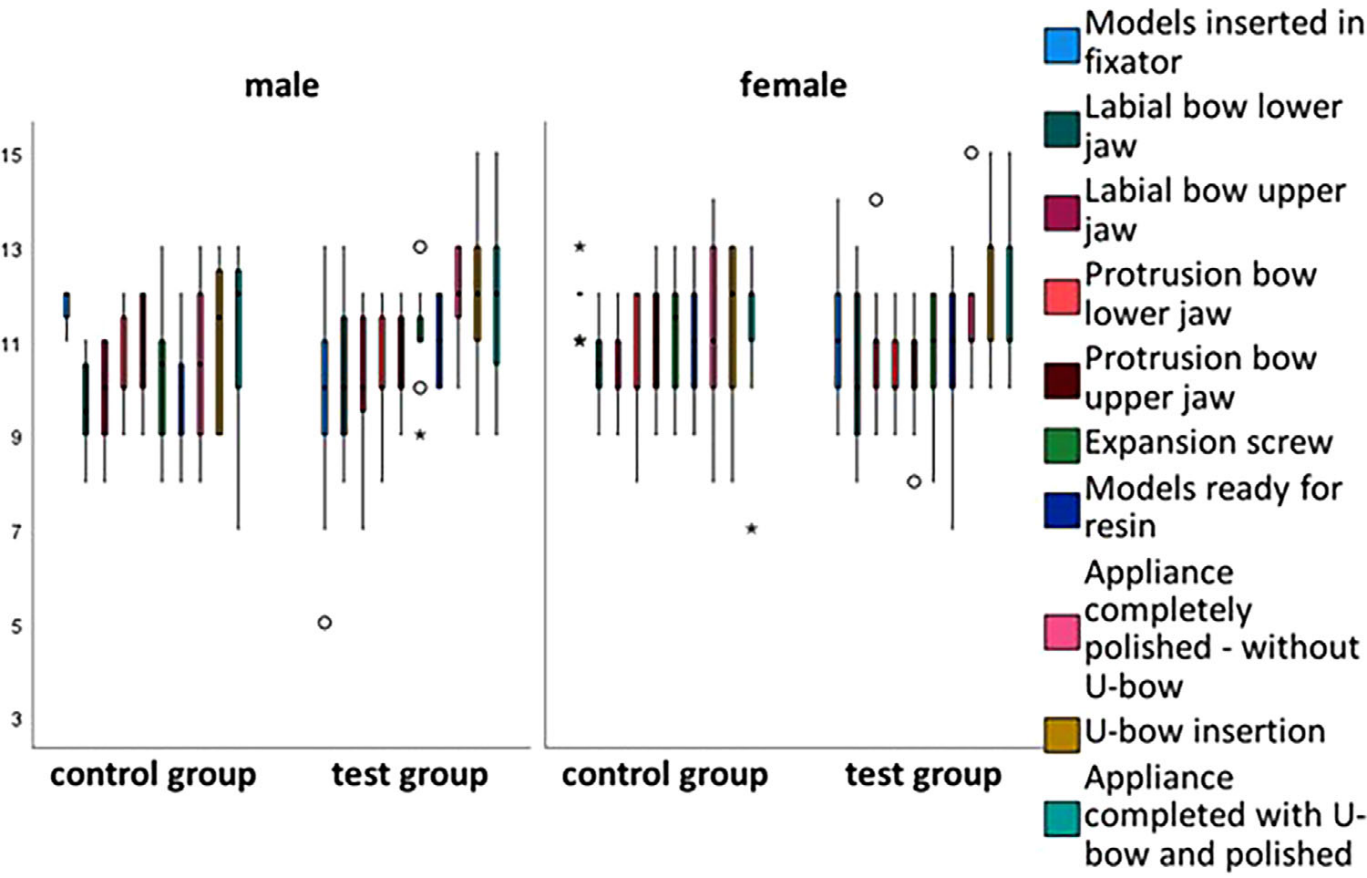
Grades of Karwetzky's U‐bow activator type 1 fabricated by the students in course 2.

**FIGURE 5:**
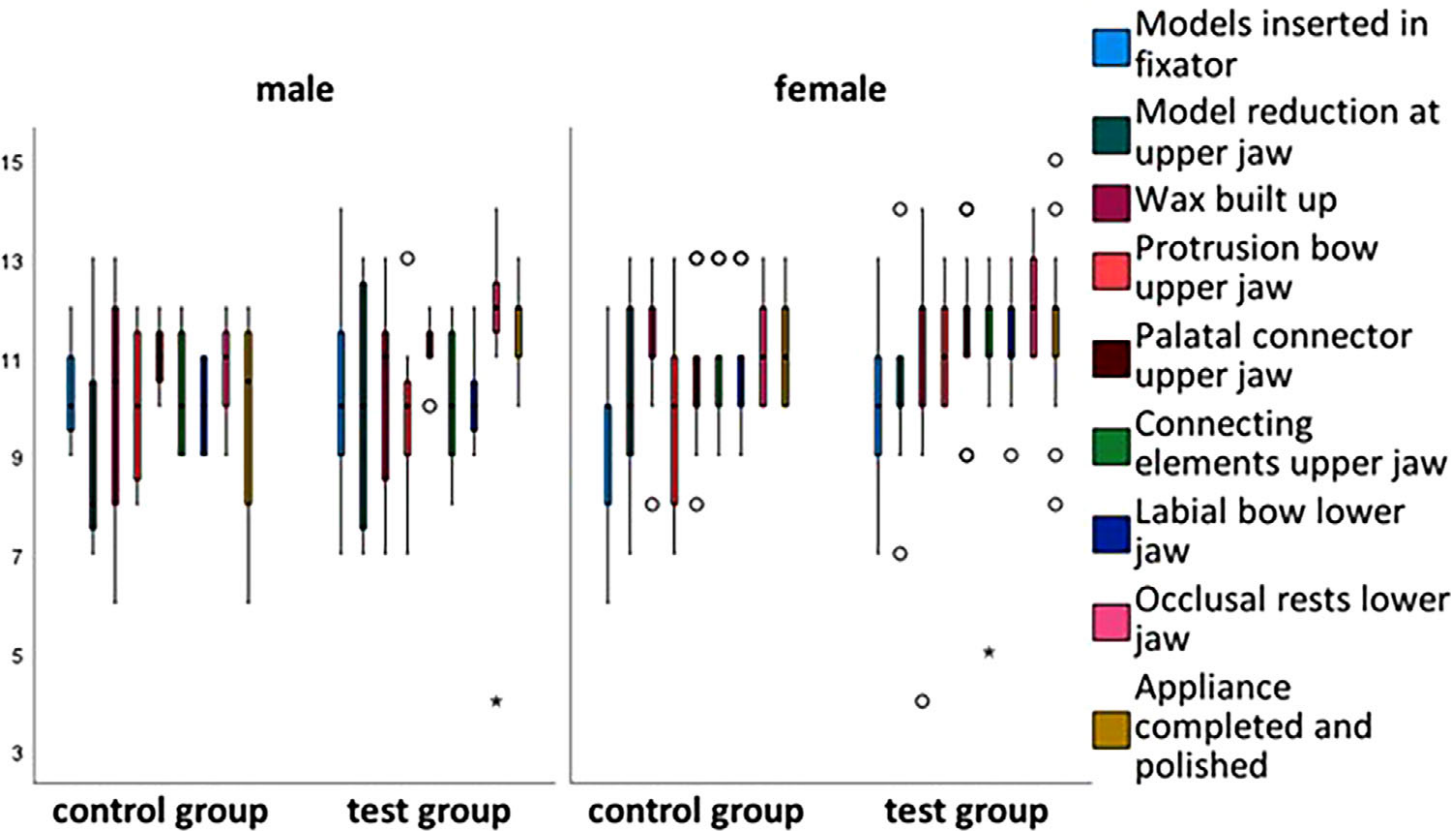
Grades of Fränkel's functional regulator type 3 fabricated by the students in course 3.

Box plots were used to show the differences in scores for the fabrication steps between the control and test groups by gender (Figures 3, 4, 5). Figure 3 shows that the majority of fabrication steps in course 1 were rated better in the test group, especially the construction plan of elements. However, there were also production steps that were rated better in the control group. In courses 2 and 3, there are more values showing better results for the test group than for the control group. Specifically, in course 2 (Figure 4), there are better results for the U‐bow insertion in the test group, whereas in course 3 (Figure 5), there are better results for the protrusion bow, the palatal connector, and the occlusal rests in the test group compared with the control group.

Regarding gender, female participants performed better than males in all three courses (*p* = 0.0207; Table 5).

**TABLE 5:** Statistical comparison for each course and in total according to gender and divided by control and test groups.

							*p* value				
	Total		Control group		Test group		Control vs test group			Male vs female	
	Male	Female	Male	Female	Male	Female	Male	Female	Male vs female	Control group	Test group
Course 1											
n	17	39	4	24	13	15	0.1398	0.7724	0.0635	0.0352*	0.3674
Mean (SD)	11.26 (0.88)	11.76 (0.83)	10.69 (0.82)	11.74 (0.94)	11.44 (0.85)	11.79 (0.64)					
Median (Range)	11.50 (9.75–12.38)	11.75 (9.38–13.25)	10.56 (10.00–11.63)	11.81 (9.38–13.25)	11.75 (9.75–12.38)	11.50 (10.75–12.88)					
Course 2											
n	19	23	8	14	11	9	0.3207	0.8497	0.3051	0.1927	0.7319
Mean (SD)	10.75 (0.84)	11.03 (0.79)	10.53 (0.92)	11.04 (0.73)	10.91 (0.78)	11.01 (0.94)					
Median (Range)	10.90 (9.20–12.00)	11.10 (9.30–12.50)	10.90 (9.20–11.70)	11.20 (9.30–12.20)	11.00 (9.70–12.00)	11.00 (9.40–12.50)					
Course 3											
n	13	27	6	14	7	13	0.6678	0.0239*	0.4185	0.8688	0.3411
Mean (SD)	10.29 (1.11)	10.65 (1.16)	10.05 (1.03)	10.17 (1.07)	10.49 (1.23)	11.16 (1.07)					
Median (Range)	10.11 (8.44–12.00)	10.56 (8.63–13.44)	10.11 (8.88–11.33)	10.06 (8.63–12.44)	10.78 (8.44–12.00)	11.22 (8.89–13.44)					
In total											
n	49	89	18	52	31	37	0.0309*	0.3707	0.0207*	0.0129*	0.2001
Mean (SD)	10.81 (0.99)	11.23 (1.05)	10.40 (0.92)	11.13 (1.12)	11.04 (0.97)	11.38 (0.93)					
Median (Range)	10.90 (8.44–12.38)	11.38 (8.63–13.44)	10.51 (8.88–11.70)	11.30 (8.63–13.25)	11.00 (8.44–12.38)	11.38 (8.89–13.44)					

## 4 DISCUSSION

This study aimed to assess the influence of a 15‐point grading system compared with a pass/fail evaluation on the quality of orthodontic appliances in dental education. Based on the results of this study, the quality of orthodontic appliances using a 15‐point grading system was slightly higher in all three courses. However, this difference was statistically significant and clinically meaningful only in one of the three courses. As a secondary outcome, female participants outperformed males in the quality of the orthodontic appliances.

These findings are supported by the results of Ba‐Ali et al.^6^ who showed that a grading system led to better student results. Our results also support the thesis that a detailed grading system makes it easier to identify weaknesses in certain areas.^2^ It should be noted that in our study, a statistically significant improvement in appliance quality associated with the 15‐point grading system was found only in one of the three courses. Possible explanations could be that students in course 3 were closer to graduation and could be better motivated by detailed grades, or the presumed increased complexity of the production steps for the Fränkel appliance, which could more accurately reveal differences between the two grading systems.

However, there seems to be a trend in the scientific literature toward the pass/fail system with authors showing that a pass/fail system does not affect the students’ performance.^7^, ^11^ A pass/fail system could also reduce competition among students and promote intrinsic motivation, which is considered the key to self‐directed life‐long learning.^11^ There is evidence that this autonomous, intrinsic motivation can lead to better grades.^13^ However, negative feedback through grades does not necessarily reduce intrinsic motivation.^14^ Deci et al.^15^ found that verbal rewards increased intrinsic motivation, whereas tangible rewards (such as trophies or awards) undermined intrinsic motivation. While most studies support the pass/fail system and express that grades do not seem to be necessary or beneficial, it seems that giving students feedback in terms of a grade gives them the opportunity to rank and judge their own performance against an objective scale or against peers and to decide for themselves how much harder they want to try and improve their own work.

It should be noted that most studies compare the two assessment systems only on theoretical skills and not on manual skills. Manual skills and expertise seem to be more difficult to measure in medical education, as there are not many studies on practical skills.^16^ It may also be that manual skills are more difficult to develop and support than intellectual skills. For example, the use of tablet computers in education can be recommended^17^ and has a positive effect on theoretical knowledge, but not on manual dexterity.^18^ Therefore, further research on the teaching of manual dexterity would be useful for the improvement of dental and medical education.

In our study, there was a gender imbalance with 89 female and 49 male participants. Notably, in all courses and in both groups in our study, the female participants performed slightly better than the male participants. Internationally, the proportion of women in dental education has increased in recent decades, although the acceptance rate for female applicants relative to the number of applicants is almost as high as for male applicants.^19^ Interest in health sciences, but also responsibilities and working conditions, seem to play a role in the decision to choose a career in dentistry.^19^ In a study by Cortright et al.,^20^ female students showed positive attitudes toward intellectual development, intrinsic motivation, and performance, while male students had negative attitudes in these areas. It is interesting to note, however, that a significant difference was found between males and females in the control group, where the evaluation took place in secret and therefore could not increase motivation. In terms of manual dexterity, hand function in women, as assessed by the Motor Performance Series, was significantly better than in men in the majority of tests, especially in the dominant hand.^21^ The results of another study showed that women had better finger movement control than men in a task that did not require object interaction or manipulation,^22^ which could explain why the female participants in our study performed slightly better than the male participants. It may be interesting to investigate this aspect in further studies.

### 4.1 Strengths and limitations

A strength of the present study is the randomized controlled trial design. Random assignment of participants helps control for potential confounding variables and increases internal validity. Furthermore, with 139 dental students participating, the study has a relatively large sample size, which increases generalizability. The use of a 15‐point grading system can be considered a simple method of providing a detailed and quantitative measure of students’ performance and is common in German general education. Using calibrated dentists to assess students’ performance increases the reliability of the assessment process. The calibration ensures consistency among raters, reducing interrater variability.

However, despite efforts to calibrate dentists, there may still be subjectivity in the grading process, which must be considered a limitation of the present study. Different dentists may interpret and apply grading criteria differently, leading to potential bias. To avoid this bias, the course supervisors decided on rating criteria for the respective orthodontic appliances prior to the start of the trial. This should be considered when interpreting the results. The use of a digital education platform could influence the dynamics of student engagement and interaction with the raters compared with a traditional paper‐based evaluation system. However, participants in both groups were provided with an iPad and received detailed instructions within the software. Only the way in which participants were informed about their performance differed between the groups. Furthermore, students were allowed to improve their work if the respective manufacturing step was not sufficient. On the one hand, this gives students the opportunity to improve their work and can reduce stress, but on the other hand, it can lead to students showing intermediate steps before they achieve the best possible result. This could lead to a bias in students' actual manual dexterity. Another limitation can be seen in the gender imbalance, which may limit the generalizability of the gender‐related findings. The study was powered for the primary outcome, which may limit the clinical implications of the subgroup analyses. However, these analyses seem valuable for understanding potential benefits and challenges specific to subpopulations and may generate hypotheses for future research. The study was conducted at a single department in Germany and focused on manual skills in orthodontic education. Therefore, findings may not be directly applicable to other fields of medical education in different cultural contexts. Further research involving larger samples and multiple institutions would improve understanding of the validity of these results and explore their clinical implications.

Students’ satisfaction and long‐term effects were not investigated in the present study, leaving room for further research. Lastly, while a statistically significant difference was found in course 3, the practical significance of this difference may be limited. Students in course 3 were closer to graduation, which could affect motivation levels, and the complexity of the production steps could differ between the courses, which is considered another limitation of this study. The small difference in mean grading values may not necessarily translate into a clinically significant improvement in the quality of orthodontic appliances.

## 5 CONCLUSION

The use of a 15‐point grading system has a positive impact on the quality of appliances in orthodontic education and can be recommended. However, the differences between 15‐point and pass/fail grading systems were small and clinically meaningful in only one of the three courses evaluated.

## AUTHOR CONTRIBUTIONS

Marina Julia Bialas, Thomas Stamm, and Moritz Blanck‐Lubarsch devised the study concept and designed the study. Marina Julia Bialas and Moritz Blanck‐Lubarsch performed data collection. Marina Julia Bialas, Jonas Q. Schmid, and Moritz Blanck‐Lubarsch analyzed the data. Marina Julia Bialas, Moritz Blanck‐Lubarsch, and Jonas Q. Schmid prepared the figures and tables. Marina Julia Bialas, Jonas Q. Schmid, and Moritz Blanck‐Lubarsch wrote the main part of the manuscript. Claudius Middelberg and Thomas Stamm contributed to writing the paper. All authors critically read, edited, and approved the final manuscript.

## CONFLICT OF INTEREST STATEMENT

The authors declare no conflict of interest.

## ETHICS STATEMENT AND CONSENT TO PARTICIPATE

Permission to conduct this study was given by the Ethics Commission of the Medical Faculty of the University of Münster, Germany (registration number: 2017‐216‐f‐S, date of approval: April 28^th^, 2017). Participation in the study was voluntary and all participants gave informed consent before participating.

## CONSENT FOR PUBLICATION

The participants gave informed consent to have their information published without identifying data.

## TRIAL REGISTRATION

The study was registered in the German Clinical Trials Register (DRKS00012672).

## Data Availability

All data generated or analyzed during this study are included in this published article.
